# Epidemiology of patients harboring carbapenemase-producing bacteria and comparison with patients with detection of extended-spectrum beta-lactamase–producing Enterobacterales—A retrospective cohort study

**DOI:** 10.1017/ice.2023.125

**Published:** 2023-12

**Authors:** Isabelle Vock, Lisandra Aguilar-Bultet, Daniel Goldenberger, Silvio Ragozzino, Sabine Kuster, Sarah Tschudin-Sutter

**Affiliations:** 1 Division of Infectious Diseases and Hospital Epidemiology, University Hospital Basel, University Basel, Switzerland; 2 Division of Bacteriology and Mycology, University Hospital Basel, University Basel, Switzerland; 3 Department of Clinical Research, University Hospital Basel, University Basel, Switzerland

## Abstract

**Objective:**

We evaluated the epidemiology of carbapenemase-producing bacteria (CPB) in Switzerland by comparing risk factors between patients colonized with CPB and patients colonized with extended-spectrum β-lactamase–producing Enterobacterales (ESBL-PE).

**Methods:**

This retrospective cohort study was conducted at the University Hospital Basel in Switzerland. Hospitalized patients with CPB in any sample between January 2008 and July 2019 were included. The ESBL-PE group consisted of hospitalized patients with detection of ESBL-PE from any sample between January 2016 and December 2018. Comparisons of risk factors for acquisition of CPB and ESBL-PE were performed by logistic regression.

**Results:**

Inclusion criteria were met for 50 patients in the CPB group and 572 in the ESBL-PE group. In the CPB group, 62% had a travel history and 60% had been hospitalized abroad. When comparing the CPB group to the ESBL-PE group, hospitalization abroad (odds ratio [OR], 25.33; 95% confidence interval [CI], 11.07–57.98) and prior antibiotic therapy (OR, 4.76; 95% CI, 2.15–10.55) remained independently associated with CPB colonization. Hospitalization abroad (*P* < .001) and prior antibiotic therapy (*P* < .001) predicted CPB in the comparison of CPB with ESBL *Escherichia coli*, whereas hospitalization abroad was associated with CPB in comparison to ESBL *Klebsiella pneumoniae*.

**Conclusions:**

Although CPB still seem to be mainly imported from areas of higher endemicity, local acquisition of CPB is emerging, especially in patients with close and/or frequent contact with healthcare services. This trend resembles the epidemiology of ESBL *K. pneumoniae*, supporting mainly healthcare-associated transmission. Frequent evaluation of CPB epidemiology is required to improve detection of patients at risk of CPB carriage.

Carbapenemase-producing bacteria (CPB) are rapidly emerging worldwide but are still infrequent in Switzerland. Since 2013, ANRESIS (the Swiss Center for Antibiotic Resistance) has reported increasing numbers of CPB; cases have more than doubled from 250 reported CPB isolates in 2013 to 542 cases in 2021.^1^ The main CPB species are *Klebsiella pneumoniae* (26%) and *Enterobacter* spp (18%), whereas the most frequently detected carbapenemases are oxacillinase (OXA) genotypes, followed by *K. pneumoniae* carbapenemases (KPC).^1,2^ Travel or migration from countries with higher endemicity is still considered the main pathway of introduction into regions of low endemicity where CPB are mainly recovered in the context of healthcare-acquired infections.^2,3^ Duration of CPB carriage is usually unknown and spontaneous decolonization is frequent, yet CPB carriage might persist up to several months to years,^4^ with higher risk of prolonged carriage in immunocompromised patients.^5^ Limited treatment options for infections caused by CPB represent a major challenge for healthcare systems, possibly increasing morbidity, mortality, and healthcare costs.^4,6^

In contrast, extended-spectrum β-lactamase–producing Enterobacterales (ESBL-PE) have reached endemic levels in the Swiss population.^1^ Predictive patient-related variables have been described for patients harboring CPB and ESBL-PE, respectively. These analyses indicated similar exposures and risk factors, such as travel to areas of high endemicity, recent hospitalization, comorbidities, invasive procedures, or previous antibiotic therapy. All of these factors impede decision making for patients at risk of CPB carriage.^3,7–12^

We analyzed characteristics of patients colonized with CPB in a tertiary-care center in a setting of low endemicity. We compared patient-related risk factors between patients colonized with CPB and patients colonized with ESBL-PE to gain further insights into the epidemiology of these high-priority antibiotic-resistant pathogens and to facilitate early detection of patients at risk. These insights will help guide empirical antibiotic treatment and correct implementation of pre-emptive infection control measures.

## Methods

### Setting and participants

This retrospective cohort study was conducted at the University Hospital Basel (USB), Switzerland, an academic tertiary-care center. The microbiology laboratory at USB identified patients harboring CPB between January 2008 and July 2019. Eligibility criteria were age ≥18 years, hospitalization in the USB and detection of at least 1 species of CPB from any sample within the respective hospitalization. The institutional infection prevention and control guidelines require all patients admitted directly from acute-care settings abroad or with a history of CPB colonization to be screened on admission. In addition, colonization with ESBL-PE and/or CPB is screened for inpatients requiring at least 24 hours of intensive care or admitted to the hematological transplant unit. A cohort of patients with ESBL-PE detected between January 2016 and December 2018 was used for comparisons of risk factors and exposures between patients colonized with CPB and ESBL-PE. Hospitalized patients aged ≥18 years with detection of ESBL-PE from any sample within the corresponding hospitalization, identified by the microbiology laboratory, were included in the ESBL-PE group. Data regarding patient selection and characteristics of the ESBL-PE cohort were previously published in the ESBL Infect study.^13^ In both cohorts, patients were excluded if refusal of subsequent use of their data was documented using the general informed consent tool of the USB.

This study was approved by the local ethics committee (Ethikkomission Nordwest- und Zentralschweiz (EKNZ), project-IDs 2019-01548 and 2017-00100). We adhered to the “Strengthening the Reporting of Observational Studies in Epidemiology” guidelines.^14^

### Study data collection and definitions

Patient data were collected from electronic medical records, anonymized, and entered into a secure REDCap (Research Electronic Data Capture) database.^15,16^ Data included (1) demographics; (2) clinical characteristics such as comorbidities, vascular hardware or urinary catheterization, recent surgeries, active open wounds, history of allogeneic stem-cell or solid-organ transplantation; (3) current treatment (concomitant medication, antibiotic therapy, immunosuppressive therapy); and (4) microbiological data. Definitions are provided in the Supplementary Material (online).

### Microbiological analyses

Both CPB and ESBL-PE were identified using standard diagnostic approaches. Identification methods of ESBL-PE were previously described in the ESBL-Infect study.^13^ Testing for CPB changed during the study period. In principal, phenotypic testing was increasingly replaced with genotypic testing according to the increasing availability of respective assays. Most recently, screening was performed using chrom ID Carba Smart agar (bioMérieux, Marcy-l’Étoile, France). After species identification, Enterobacterales were tested for carbapememase genes using the eazyplex Superbug CRE panel (easyplex, Gars-Bahnhof, Germany) detecting KPC, VIM, NDM, OXA-48, and OXA-181. If phenotypic testing suggests carbapenem resistance and genotypic screening is negative, further phenotypic testing is performed using the Mastdiscs combi Carba plus test (Mast Group, Reinfeld, Germany). *Pseudomonas* spp were analyzed with GeneXpert Carba-R (Cepheid Switzerland, Thalwil, Switzerland) detecting VIM, IMP-1, NDM, KPC, and OXA-48. *Acinetobacter baumannii*–producing carbapenemases were identified using the eazyplex SuperBug complete A test system, which includes KPC, VIM, OXA-48, OXA-23, OXA-40, and OXA-58.

### Statistical analyses

Sample size estimation was fixed based on identification of eligible patients by the microbiology laboratory within the study periods. Missing data were considered as the absence of an assessed factor. Descriptive analyses were summarized as counts and proportions for categorical variables, using the Fisher's exact test for comparisons. Medians and interquartile ranges of continuous variables were analyzed using the Mann-Whitney *U* test, as the Shapiro-Wilk revealed all continuous variables to be abnormally distributed. Univariable and multivariable regression analyses were applied for comparisons of the CPB group versus the ESBL-PE group as well as subgroup analyses of the CPB group. The Hosmer-Lemeshow goodness-of-fit test was applied to assess model fit (insignificant *P* values indicated adequate fit). We performed sensitivity analyses (1) excluding patients in the CPB group with nonfermenters and (2) excluding patients in the CPB group with nonfermenters and Enterobacterales not recovered during the same timeframe as patients colonized with ESBL-PE. Statistical significance was set at *P* < .05. Statistical analyses were performed using STATA version 16.1 software (StataCorp, College Station, TX).

## Results

Within the study period, inclusion criteria were met by 50 patients in the CPB group and by 572 patients in the ESBL-PE group. The baseline characteristics, exposures and microbiological data of both the CPB and the ESBL-PE cohorts are presented in Table 1 and Supplementary Table 1 (online).

**Table 1.** Baseline Characteristics of the CPB Group and the ESBL-PE Group

Characteristic	CPB Group (n=50)		ESBL-PE Group (n=572)		*P* Value
	No. or Median	% or IQR	No. or Median	% or IQR	
**Demographics and clinical data**					
Sex, female	24	48.0	277	48.4	.954
Age, median y	63	53–75	70	56–81	.090
**Admission from**					**<.001**
Home	25	50.0	401	70.1	
Nursing home	2	4.0	69	12.1	
Other acute healthcare facility	23	46.0	99	17.3	
Unknown	0		3	0.5	
**Ward**					**<.001**
Medical	17	34.0	332	58.0	
Surgery	28	56.0	188	32.9	
Other	5	10.0	52	9.1	
Stay in ICU within the index hospitalization	22	44.0	144	25.2	**.004**
Length of hospital stay, d	22	9–42	13	7–23	**.002**
Death	9	18.0	32	5.6	**.001**
History of hospitalization^a^	42	84.0	396	69.2	**.028**
History of ICU stay^a^	10	20.0	87	15.2	.371
Travel history^a^	31	62.0	89	15.6	**<.001**
Hospitalization abroad^a^	30	60.0	35	6.12	**<.001**
Charlson comorbidity index	1	1–2	2	0–3	.692
Open wounds	9	18.0	66	11.5	.178
Recent surgery^a^	27	54.0	160	28.0	**<.001**
Urinary catheterization^b^	21	42.0	173	30.2	.085
Indwelling vascular hardware^c^	4	8.0	22	3.9	.159
Dialysis	1	2.0	10	1.8	.897
Solid-organ transplantation	0	0	28	4.9	.109
Allogeneic stem-cell transplantation	1	2.0	16	2.8	.740
History of colonization with CPB or ESBL-PE^a,d^	12	24.0	237	41.4	**.016**
Recent antibiotic therapy^e^	39	78.0	279	48.8	**<.001**
Duration of antibiotic therapy, d	9.5	4-23	13	6-32	.193
Immunosuppressive therapy^a^	10	20.0	154	26.9	.287
Proton-pump inhibitor^e^	22	44.0	317	55.4	.120

Note. CPB, carbapenemase-producing bacteria; ESBL-PE, extended-spectrum β-lactamase–producing Enterobacterales; IQR, interquartile range; ICU, intensive care unit; MRSA, methicillin-resistant *Staphylococcus aureus*; VRE, vancomycin-resistant enterococci. Bold indicates statistical significance.

a Within the previous 12 mo.

b Within 30 d prior to CPB detection.

c To be in place for at least 7 d prior to CPB detection.

d refers to ESBL-PE and/or CPB in the CPB group and to only ESBL-PE in the ESBL-PE group.

e Within 3 mo prior to CPB detection.

### CPB group

Overall, 42 patients (84.0%) had a history of hospitalization within the previous 12 months. Among 31 patients (62.0%) travelling outside Switzerland within the prior 12 months, 30 patients had been hospitalized abroad. For 6 patients (12.0%) neither exposure to healthcare settings nor travel abroad was recorded. Antibiotic therapy within 3 months prior to the index hospitalization was administered in 39 patients (78.0%). Among 33 patients (66.0%) screened for multidrug-resistant bacteria at hospital admission, co-colonization with ESBL-PE was detected in 6 patients. First detection of CPB within the hospitalization derived mainly from clinical samples (26 patients, 52.0%), followed by admission screening samples (16 patients, 32.0%) and consecutive screening samples (8 patients, 16.0%). This screening was performed routinely in high-risk wards such as the intensive care unit (ICU) and the hematologic ward. In patients with a history of hospitalization abroad, first detection of CPB derived from admission screenings in 10 cases (33.3%), from consecutive screenings in 4 cases (13.3%), and from clinical samples in 16 cases (53.3%).

### ESBL-PE group

Travel history outside Switzerland was positive in 15.6% of patients, and 48.8% had received antibiotic medication in the prior 3 months.

### Subgroup analyses of CPB patients according to travel history

A subgroup analysis comparing patients of the CPB group with (n = 31) versus without (n = 19) travel history was performed (Supplementary Table 2 online). Admission from other acute-care facilities (OR, 3.88; 95% CI, 1.12–13.47; *P* = .033) and a history of hospitalization (OR, 6.69; 95% CI, 1.19–37.71; *P* = .031) were associated with the travel subgroup. A history of CPB/ESBL-PE colonization (OR, 0.20; 95% CI, 0.05–0.82; *P* = .025) and recent immunosuppressive therapy (OR, 0.18; 95% CI, 0.04–0.83; *P* = .028) were associated with the CPB nontraveling subgroup. Microbiological differences of CPB species and carbapenemases genotypes between CPB travel versus nontravel subgroups are shown in Supplementary Table 3 (online). *Acinetobacter baumannii* and OXA-type carbapenemases were more frequently recovered in patients with a travel history than without.

**Table 2.** Comparison of Risk Factors Between Patients With Detection of CPB Versus Patients With Detection of ESBL-PE

Characteristic	Univariable Analysis		Multivariable Analysis	
	OR (95% CI)	*P* Value	OR (95% CI)	*P* Value
**Demographics and clinical data**				
Sex, female	0.98 (0.55–1.75)	.954		
Age	0.99 (0.96–1.00)	.199		
Ward	0.80 (0.54–1.17)	.244		
History of hospitalization^a^	2.33 (1.07–5.07)	**.032**	0.54 (0.20–1.44)	.214
History of ICU stay^a^	1.39 (0.67–2.89)	.373		
Hospitalization abroad^a^	23.01 (11.90–44.58)	**<.001**	25.33 (11.07–57.98)	**<.001**
Charlson comorbidity index	0.95 (0.82–1.19)	.469		
Open wounds	1.68 (0.78–3.62)	.183		
Surgery^a^	3.00 (1.68–5.43)	**<.001**	1.87 (0.93–3.78)	.081
Urinary catheterization^b^	1.67 (0.93–3.01)	.088		
Vascular hardware^c^	2.17 (0.72–6.58)	.169		
Dialysis	1.14 (0.14–9.15)	.897		
Allogeneic stem-cell transplantation	0.71 (0.09–5.46)	.741		
History of colonization with ESBL-PE/CPB^a,d^	0.45 (0.22–0.87)	**.018**	0.90 (0.39–2.06)	.804
History of infection with ESBL-PE/CPB^a,d^	0.42 (0.15–1.18)	.099		
History of antibiotic therapy^e^	3.72 (1.87–7.42)	**<.001**	4.76 (2.15–10.55)	**<.001**
Duration of antibiotic therapy, d	1.00 (1.00–1.01)	.130		
Immunosuppressive therapy^a^	0.68 (0.33–1.39)	.289		
Proton pump inhibitor^e^	0.63 (0.35–1.13)	.122		
**Outcome**				
Length of hospital stay	1.01 (1.00–1.01)	**.015**		
Survival	0.26 (0.12–0.60)	**.001**		
ICU	2.33 (1.30–4.21)	**.005**		

Note. CPB, carbapenemase-producing bacteria; ESBL-PE, extended-spectrum β-lactamase–producing Enterobacterales; OR, odds ratio; CI, confidence interval, ICU, intensive care unit. Bold indicates statistical significance.

a Within the past 12 mo.

b Within 30 d prior to CPB detection.

c To be in place for at least 7 d prior to CPB detection.

d Refers to ESBL-PE and/or CPB in the CPB group and to only ESBL-PE in the ESBL-PE group.

e Within 3 mo prior to CPB detection.

### Comparison of the CPB group with the ESBL-PE group

Hospitalization abroad, recent surgery, and history of antibiotic therapy within the prior 3 months were associated with carriage of CPB in univariable analysis. History of colonization with CPB and/or ESBL-PE was associated with detection of ESBL-PE. After multivariable analysis, hospitalization abroad and history of antibiotic therapy remained associated with detection of CPB (Hosmer Lemeshow χ^2^, 5.65; *P* = .582) (Table 2).

Detection of CPB was associated with a longer duration of hospital stay and a lower survival rate (Table 2).

Subgroup analyses of the CPB cohort versus patients of the ESBL-PE cohort colonized with only *E. coli* and only *K. pneumoniae* were performed additionally.

Multivariable analyses of the CPB group versus the ESBL *E. coli* group revealed hospitalization abroad and history of antibiotic therapy to be associated with the CPB group (Hosmer Lemeshow χ^2^, 7.11; *P* = .525), whereas the only variables associated with the CPB group in the comparison with the ESBL *K. pneumoniae* group were female sex and hospitalization abroad (Hosmer-Lemeshow χ^2^, 7.74; *P* = .459) (Table 3).

**Table 3.** Subgroup Analysis of Patients With Detection of CPB Versus Patients With Detection of Only ESBL *E. coli* (A) and Versus Patients With Detection of Only ESBL *K. pneumoniae* (B)

	(A) CPB vs ESBL *E. coli* (n = 467)				(B) CPB vs ESBL *K. pneumoniae* (n = 65)			
	Univariable Analyses		Multivariable Analyses		Univariable Analyses		Multivariable Analyses	
	OR (95% CI)	*P* Value	OR (95% CI)	*P* Value	OR (95% CI)	*P* Value	OR (95% CI)	*P* Value
Sex, female	0.86 (0.48–1.53)	.608			2.60 (1.19–5.71)	**.017**	2.94 (1.10–7.87)	**.032**
Age	0.99 (0.97–1.00)	.098			1.00 (0.98–1.02)	.834		
Location prior to admission^a^	1.00 (0.95–1.04)	.904			1.64 (1.10–2.45)	**.017**	1.41 (0.82–2.43)	.220
Ward	0.76 (0.52–1.12)	.176			1.12 (0.73–1.71)	.605		
History of hospitalization^b^	2.68 (1.23–5.86)	**.013**	0.58 (0.22–1.51)	.265	0.74 (0.26–2.12)	.572		
History of ICU stay^b^	1.83 (0.87–3.87)	.111			0.49 (0.21–1.16)	.104		
Hospitalization abroad^b^	32.85 (15.58–65.14)	**<.001**	37.25 (15.74–88.15)	**<.001**	10.69 (4.21–27.13)	**<.001**	6.19 (2.08–18.48)	**.001**
Charlson comorbidity index	0.95 (0.82–1.10)	.457			0.93 (0.75–1.14)	.464		
Active open wounds	1.83 (0.84–3.99)	.128			1.21 (0.45–3.24)	.708		
Surgery^b^	3.47 (1.92–6.29)	**<.001**	2.02 (0.94–4.36)	.072	1.76 (0.84–3.71)	.137		
Urinary catheterization^c^	1.82 (1.00–3.30)	**.049**	1.12 (0.51–2.43)	.782	1.02 (0.48–2.15)	.960		
Vascular hardware^d^	2.81 (0.89–8.90)	.078			0.86 (0.23–3.21)	.817		
Dialysis	0.93 (0.12–7.44)	.948			–			
Allogeneic stem cell transplantation	0.71 (0.09–5.57)	.747			0.64 (0.06–7.30)	.721		
History of colonization with CPB/ESBL-PE^b,e^	0.52 (0.26–1.02)	.056			0.22 (0.10–0.51)	**<.001**	0.57 (0.20–1.61)	.289
History of infection with CPB/ESBL-PE^b,e^	0.45 (0.16–1.30)	.141			0.23 (0.07–0.72)	**.012**		
History of antibiotic therapy^f^	4.16 (2.08–8.31)	**<.001**	5.98 (2.49–14.33)	**<.001**	2.22 (0.96–5.11)	.062		
Duration of antibiotic therapy, d	1.01 (1.00–1.01)	.149			1.00 (1.00–1.01)	.807		
Immunosuppressive therapy^b^	0.67 (0.33–1.38)	.276			0.61 (0.25–1.45)	.261		
Proton pump inhibitor^f^	0.72 (0.40–1.29)	.269			0.32 (0.15–0.70)	**.004**	0.42 (0.16–1.09)	.075
**Outcome**								
ICU stay	2.55 (1.40–4.64)	**.002**			1.18 (0.56–2.49)	.666		
Length of hospital stay	1.01 (1.00–1.02)	**.004**			1.00 (0.99–1.01)	.577		
Survival	0.28 (0.12–0.63)	**.002**			0.38 (0.12–1.21)	.103		

Note. CPB, carbapenemase-producing bacteria; ESBL-PE, extended-spectrum β-lactamase–producing Enterobacterales; OR, odds ratio; CI, confidence interval; ICU, intensive care unit. Bold indicates statistical significance.

a Home, other acute-care facility or nursing home.

b Within the previous 12 mo.

c Within 30 d prior to CPB detection.

d To be in place for at least 7 days prior to CPB detection.

e Refers to ESBL-PE and/or CPB in the CPB group and to only ESBL-PE in the ESBL-PE group.

f Within 3 mo prior to CPB detection.

### Sensitivity analyses

Both sensitivity analyses (only considering patients a) colonized with carbapenemase-producing Enterobacterales and b) detected between January 2016 and December 2018), confirmed both hospitalization abroad and history of antibiotic therapy being associated with colonization with CPB compared to ESBL-PE (Supplementary Tables 4 and 6 online) and compared to ESBL *E. coli* (Supplementary Tables 5 and 7 online). Hospitalization abroad was inconsistently associated with colonization with CPB compared to ESBL *K. pneumoniae*. Both sensitivity analyses revealed that exposure to proton-pump inhibitors (PPIs) differed between these 2 groups (Supplementary Tables 5 and 7 online).

## Discussion

In a setting of low endemicity with only sporadic cases of CPB, colonization with CPB could be related to travel abroad in 62% of all patients and any previous hospitalization in 84% of all patients. Moreover, 38% of patients were likely to have acquired colonization in Switzerland. Of concern, no exposure to healthcare settings or travel abroad was recorded for 12% of patients, pointing to potential community CPB transmission. Hospitalization abroad and recent antibiotic therapy were associated with increased risk of CPB in our setting of low CPB endemicity compared to patients with detection of ESBL-PE. The epidemiology of ESBL *K. pneumoniae* seems more comparable to the epidemiology of CPB rather than ESBL *E. coli* in terms of antibiotic exposure. This finding highlights the importance of selection pressure in addition to exposure in healthcare settings for successful colonization with both CPB and ESBL *K. pneumonae* compared with ESBL *E. coli*.

An analysis of our CPB cohort suggests 2 different epidemiologic characteristics. One comprises patients with travel history within the past 12 months, accounting for 2/3 of our study population. Furthermore, these patients were frequently in contact with a foreign healthcare environment, supporting probable introduction of CPB into Switzerland and hereby screening recommendations for repatriated patients or patients with known hospitalization abroad in the prior months.^17^ Interestingly, recent local data documented stabilization in CPB rates in 2020–2021, which might reflect lower travel activities due to the COVID-19 pandemic.^18^

The second epidemiologic subgroup comprised patients without travel history, who were more likely to have a history of CPB or ESBL-PE colonization and/or infection within the past year and were more likely to have been exposed to immunosuppressive therapy. This finding points to a population of patients with higher vulnerability and therefore possibly to a higher frequency of healthcare contacts. The size of this patient population is likely to increase in future decades due to medical progress and an aging population benefiting from advanced therapies. Thus, the need for constant evaluation of local CPB epidemiology on a national or even institutional level is underscored to adapt screening recommendations.

In most cases within both subgroups, CPB carriage was detected in clinical samples rather than admission or routine screening samples. Although recommendations for admission screenings exist in most Swiss hospitals, screening triggers and sampling sites vary widely among between Swiss healthcare institutions, as reported by Martischang et al.^19^ Only 115 hospitals (78%) targeted CP Enterobacterales specifically, underscoring the need to improve CPB screening strategies.

In both CPB subgroups, OXA genotypes were the most prevalent followed by KPC, which is in line with recent national^1^ as well as European data.^20,21^ However, the CPB travel subgroup was associated with a significantly higher percentage of OXA genotypes, possibly associated with an also significantly higher amount of detection of CP *A. baumannii* in the CPB travel subgroup, highlighting the emergence of OXA-producing *A. baumannii* in Mediterranean countries,^22,23^ which account for almost 65% of travel destinations in our cohort (Supplementary Table 8 online).

When comparing the CPB group to the ESBL-PE group, hospitalization abroad and previous antibiotic exposure were associated with carriage of CPB. Furthermore, these differences in epidemiology of the CPB cohort versus the ESBL-PE cohort derive mainly from comparisons to patients with detection of ESBL *E. coli*, as revealed by our subgroup analysis. Previous antibiotic exposure did not differ between patients colonized with CPB and ESBL *K. pneumoniae*. This finding highlights the importance of selection pressure in addition to exposure within healthcare settings for successful colonization compared to ESBL *E. coli* or continuing re-exposure within the community to ESBL *E. coli*. In contrast to ESBL *E. coli*, ESBL *K. pneumoniae* is more frequently related to nosocomial acquisition.^24^ Thus, infection prevention and control measures should be tailored to control transmission of ESBLs to the respective species.^25^ Female sex was related to detection of CPB rather than ESBL *K. pneumoniae* in our cohort, and PPI use was associated with ESBL *K. pneumoniae* rather than CPB in both sensitivity analyses. Both associations remain unclear at this point, especially because PPI use has been associated with an increase in risk of acquisition of both CPB and ESBL producers.^26^

This study had several limitations. Due to the retrospective, single-center design, data might not be applicable to other settings and institutions especially in settings of high endemicity. The small sample size of the CPB group might have limited the detection of further predictors. Because assessment of travel history might be biased in favor of patients with very recent stay abroad, repatriation or patients questioned for infection of unknown origin, a lack of admission screening might have led to underdetection of CPB and underestimation of the proportion of travel history in CPB-positive patients. The differing timeframes of data acquisition of the CPB group (11 years) and the ESBL-PE group (3 years) may have precluded consideration of possible changes of epidemiology. To address this shortcoming, we performed sensitivity analyses considering only the same timeframes. Last, our study was designed to specifically study the epidemiology of β-lactamases (ESBLs and carbapenemases) conferring durable resistance rather than inducible resistance-mechanisms such as efflux-pump upregulation or porin loss. To address species-related differences between Enterobacterales and nonfermenters, additional sensitivity analyses were performed.

In conclusion, this study confirms low yet increasing rates of CPB carriage in Switzerland. Although CPB is still being mainly imported from areas of higher endemicity, local acquisition of CPB is emerging, especially in patients with close and/or frequent contact with healthcare services. Thus, frequent evaluation of CPB epidemiology on national and/or institutional levels is required to improve detection of patients at risk of CPB carriage.

## Supplementary material

For supplementary material accompanying this paper visit https://doi.org/10.1017/ice.2023.125. click here to view supplementary material
