# Supplementary material for: Epidemiology of patients harboring carbapenemase-producing bacteria and comparison with patients with detection of extended-spectrum beta-lactamase–producing Enterobacterales—A retrospective cohort study
Source: Infect Control Hosp Epidemiol. 2023 Jul 10;44(12):1959–65. doi: 10.1017/ice.2023.125 (PMC10755146; doi:10.1017/ice.2023.125)
Supplement: Supplementary file 1 [file S0899823X23001253sup001.docx]

**Supplementary material**

Definitions

Comorbidities were assessed using the Charlson Comorbidity Index. Indwelling hardware was defined as vascular hardware to be in place for at least 7 days prior to CPB or ESBL-PE detection. Urinary catheterization was defined as transurethral or suprapubic catheterization within 30 days prior to detection of CPB or ESBL-PE. Recent surgeries were assessed within 12 months prior to detection of CPB or ESBL-PE. Antibiotic therapy was assessed within 3 months prior to and during the index hospitalization and immunosuppressive therapy within the prior 12 months.

A time-frame of 12 months prior to the index hospitalization was used for definition of history of colonization or infection with CPB or ESBL-PE, history of hospitalization (>1 night stay in an acute-care facility), travel history (>1 night stay outside of Switzerland) and hospitalization abroad (>1 night stay in an acute-care facility).

**Supplementary tables**

| **Supplementary table 1** Microbiological baseline data of the CPB group and the ESBL-PE group | | | | | |
| --- | --- | --- | --- | --- | --- |
|  | CPB-group (n = 50) | | ESBL-PE-group (n=572) | | p-value |
|  | N/median | %/IQR | N/median | %/IQR |  |
| Admission screening   - Negative - CPB - ESBL-PE - MRSA - VRE | 33  8  17  12  5  0 | 66.0  16.0  34.0  24.0  10.0  0 |  |  |  |
| First detection of CPB within the hospitalization   - Admission screening^a^ - Consecutive screening - Clinical samples | 16  8  26 | 32.0  16.0  52.0 |  |  |  |
| Infection caused by CPB within the corresponding hospitalization | 15 | 30.0 |  |  |  |
| Species^b^   - *A. baumannii* - *C. freundii* - *C. koseri* - *E. cloacae* - *E. coli* - *K. aerogenes* - *K. pneumoniae* - *K. oxytoca* - *K. variicola* - *P. mirabilis* - *P. stuartii* - *P. aeruginosa* - *P. alcaligenes* | 19  2  1  2  17  -  18  2  -  1  1  8  1 | 38.0  6.5  3.2  6.5  34.0  -  36.0  4.0  -  3.2  3.2  16.0  2.0 | -  1  -  5  501  1  100  -  3  2  -  -  - | -  0.2  -  0.9  87.6  0.2  17.5  -  0.5  0.4  -  -  - |  |
| Carbapenemase genotype^c^   - VIM - NDM - KPC - OXA - IMP | 7  7  7  38  2 | 14.0  14.0  14.0  76.0  4.0 |  |  |  |
| VIM = Verona Integron-Encoded Metallobetalactamase, NDM = New Delhi Metallobetalactamase, KPC = *Klebsiella pneumoniae* carbapenemase, OXA = oxacillinase, CPB = carbapenemase-producing bacteria, ESBL-PE = extended-spectrum beta-lactamase-producing Enterobacterales, MRSA = methicillin-resistant *Staphylococcus aureus*, VRE = vancomycin-resistant Enterococci, IQR = interquartile range  ^a^ for one patient admission screening was performed and positive for CPB, however first detection of CPB resulted from clinical samples taken several hours prior to screening samples  ^b^ Within the CPB group, 10 patients (20.0%) were colonized with multiple species of CPB, within the ESBL-PE group 38 patients (6.6%) were colonized with multiple species of ESBL-PE  ^c^ Within 8 patients (16.0%) multiple carbapenemases genotypes were detected | | | | | |

| **Supplementary table 2:** Subgroup analysis of patients of the CPB group with travel history versus without travel history within the past 12 months | | | | | | | | | | | |
| --- | --- | --- | --- | --- | --- | --- | --- | --- | --- | --- | --- |
|  | **Travel (n = 31)** | | | | **non-travel (n = 19)** | | | | **Univariable analysis** | | |
|  | N/ median | | %/IQR | | N/ median | | %/ IQR | | OR (95%CI) | | p-value |
| **Demographics** | | | | | | | | | | | |
| Age | 60 | 51-71 | | 70 | | 56-83 | | 0.98 (0.94-1.01) | | 0.158 | |
| Female sex | 13 | 41.9 | | 11 | | 57.9 | | 0.53 (0.17-1.67) | | 0.275 | |
| ICU stay | 16 | 51.6 | | 6 | | 31.6 | | 2.31 (0.70-7.65) | | 0.170 | |
| Admission from   - Home - Nursing home - Other acute care hospital | 12  1  18 | 38.7  3.2  58.1 | | 13  1  5 | | 68.4  5.3  26.3 | | 0.60 (0.04-10.2)  3.88 (1.12-13.47) | | 0.724  **0.033** | |
| History of hospitalization^a^ | 29 | 93.6 | | 13 | | 68.4 | | 6.69 (1.19-37.71) | | **0.031** | |
| History of ICU stay^a^ | 5 | 17.2 | | 5 | | 38.5 | | 0.33 (0.07-1.46) | | 0.144 | |
| Death   - Attributable to CPB-Infection | 5  1 | 16.1  3.2 | | 4  2 | | 21.1  10.5 | | 1.38 (0.32-5.97) | | 0.661 | |
| Length of stay (days) | 25 | 9-42 | | 21 | | 9-42 | | 0.99 (0.98-1.01) | | 0.391 | |
| **Clinical characteristics** | | | | | | | | | | | |
| Charlson Comorbidity Index | 1 | 0-2 | | 2 | | 1-4 | | 0.87 (0.62-1.21) | | 0.398 | |
| Open wounds | 4 | 12.9 | | 5 | | 26.3 | | 0.41 (0.10-1.79) | | 0.239 | |
| Recent surgery^a^ | 19 | 61.3 | | 8 | | 42.1 | | 2.18 (0.68-6.96) | | 0.190 | |
| Urinary catheterization^b^ | 15 | 48.4 | | 6 | | 31.6 | | 2.03 (0.61-6.72) | | 0.246 | |
| Vascular hardware^c^ | 2 | 6.5 | | 2 | | 10.5 | | 0.59 (0.08-4.55) | | 0.610 | |
| History of colonization with CPB/ESBL-PE^a^ | 4 | 12.9 | | 8 | | 42.1 | | 0.20 (0.05-0.82) | | **0.025** | |
| **Therapeutic data** | | | | | | | | | | | |
| Antibiotic therapy before index hospitalization^d^ | 17 | 54.8 | | 13 | | 68.4 | | 0.56 (0.17-1.86) | | 0.344 | |
| Antibiotics between hospital admission and CPB detection | 19 | 61.3 | | 10 | | 52.6 | | 1.43 (0.45-4.52) | | 0.548 | |
| Immunosuppressive therapy^a^ | 3 | 9.7 | | 7 | | 36.8 | | 0.18 (0.04-0.83) | | **0.028** | |
| Proton pump inhibitor^d^ | 11 | 35.5 | | 11 | | 57.9 | | 0.40 (0.12-1.29) | | 0.125 | |
| ICU = Intensive Care Unit, OR = odds ratio, 95%CI = 95% confidence interval, CPB = carbapenemase-producing bacteria, ESBL-PE = extended-spectrum beta-lactamase-producing Enterobacterales  ^a^ within the past 12 months  ^b^ within 30 days prior to CPB detection  ^c^ to be in place for at least 7 days prior to CPB detection  ^d^ within 3 months prior to CPB detection | | | | | | | | | | | |

| **Supplementary table 3:** Subgroup analysis of microbiological data of patients of the CPB group with travel history versus without travel history | | | | | | | |
| --- | --- | --- | --- | --- | --- | --- | --- |
|  | | **Travel**  **(n = 31)** | | **non-travel**  **(n = 19)** | | **Univariable analysis** | |
|  | N / median | | % / IQR | N / median | % / IQR | OR (95%CI) | p-value |
| **Microbiological data** | | | | | | | |
| CPB species   - *A. baumannii* - *C. freundii* - *C. koseri* - *E.cloacae* - *E. coli* - *K.pneumoniae* - *K. oxytoca* - *P. mirabilis* - *P. stuartii* - *P. aeruginosa* - *P. alcaligenes* | 17  2  1  2  8  12  1  1  1  5  0 | | 54.8  6.5  3.2  6.5  25.8  38.7  3.2  3.2  3.2  16.1 | 2  0  0  0  9  6  1  0  0  3  1 | 10.5  47.4  31.6  5.3  15.8  5.3 | 10.32 (2.03-52.52)  -  -  -  0.37 (0.12-1.29)  1.37 (0.41-4.58)  0.60 (0.04-10.20)  -  -  1.03 (0.22-4.89)  - | **0.005**  -  -  -  0.123  0.611  0.724  -  -  0.975  - |
| Carbapenemase genotype   - VIM - NDM - KPC - OXA - IMP | 2  6  4  27  2 | | 6.5  19.4  12.9  87.1  12.5 | 5  1  3  11  0 | 26.3  5.3  15.8  57.9 | 0.19 (0.03-1.12)  4.32 (0.48-39.07)  0.79 (0.16-4.00)  4.90 (1.22-19.71)  - | 0.067  0.193  0.776  **0.025**  - |
| VIM = Verona Integron-Encoded Metallobetalactamase, NDM = New Delhi Metallobetalactamase, KPC = *Klebsiella pneumoniae* carbapenemase, OXA = oxacillinase, CPB = carbapenemase-producing bacteria, ESBL-PE = extended-spectrum beta-lactamase-producing Enterobacterales  IQR = interquartile range, OR = odds ratio, 95%CI = 95% confidence interval | | | | | | | |

| **Supplementary table 4:** Comparison of risk-factors between patients with detection of carbapenemases-producing Enterobacterales (n=30) versus patients with detection of ESBL-PE (n=572) | | | | |
| --- | --- | --- | --- | --- |
|  | Univariable analysis | | Multivariable analysis | |
|  | OR (95% CI) | p-value | OR (95% CI) | p-value |
| Demographics and clinical data | | | | |
| Female sex | 1.22 (0.58-2.54) | 0.601 |  |  |
| Age | 0.99 (0.97-1.01) | 0.456 |  |  |
| Ward | 0.98 (0.64-1.50) | 0.912 |  |  |
| History of hospitalization^a^ | 1.46 (0.62-3.47) | 0.391 |  |  |
| History of ICU stay^a^ | 1.39 (0.55-3.51) | 0.481 |  |  |
| Hospitalisation abroad^a^ | 11.73 (5.28-26.09) | **<0.001** | 11.26 (4.83-26.28) | **<0.001** |
| Charlson Comorbidity Index | 1.07 (0.91-1.26) | 0.399 |  |  |
| Open wounds | 1.18 (0.40-3.49) | 0.765 |  |  |
| Surgery^a^ | 2.56 (1.23-5.39) | **0.012** | 1.56 (0.70-3.48) | 0.282 |
| Urinary catheterization^b^ | 1.15 (0.53-2.52) | 0.720 |  |  |
| Vascular hardware^c^ | 1.79 (0.40-7.98) | 0.448 |  |  |
| Dialysis | 1.94 (0.24-15.66) | 0.535 |  |  |
| Allogeneic stem cell transplantation | 1.20 (0.15-9.35) | 0.863 |  |  |
| History of colonization with ESBL-PE/CPB^a,d^ | 0.43 (0.18-1.02) | 0.055 |  |  |
| History of infection with ESBL-PE/CPB^a,d^ | 0.34 (0.08-1.46) | 0.146 |  |  |
| History of antibiotic therapy^e^ | 2.45 (1.10-5.44) | **0.028** | 2.72 (1.17-6.34) | **0.020** |
| Duration of antibiotic therapy (days) | 1.01 (1.00-1.02) | 0.112 |  |  |
| Immunosuppressive therapy^a^ | 0.99 (0.43-2.26) | 0.975 |  |  |
| Proton pump inhibitor^e^ | 0.54 (0.25-1.13) | 0.103 |  |  |
| OR = odds ratio, 95%CI = 95% confidence interval, ICU = intensive care unit, CPB = carbapenemases-producing bacteria, ESBL-PE = extended-spectrum beta-lactamase-producing Enterobacterales | | | | |
| ^a^ within the past 12 months  ^b^ within 30 days prior to CPB detection  ^c^ to be in place for at least 7 days prior to CPB detection  ^d^ refers to ESBL-PE and/or CPB in the CPB group and to only ESBL-PE in the ESBL-PE group  ^e^ within 3 months prior to CPB detection | | | | |

| **Supplementary table 5:** Subgroup analysis of patients with detection of only CP-Enterobacterales (n=30) versus patients with detection of only ESBL-*E.coli* (n=467) **(A)** and versus patients with detection of only ESBL-*K. pneumoniae* (n=65) **(B)** | | | | | | | | |
| --- | --- | --- | --- | --- | --- | --- | --- | --- |
|  | **(A)** | | | | **(B)** | | | |
|  | Univariable analyses | | Multivariable analyses | | Univariable analyses | | Multivariable analyses | |
|  | OR (95% CI) | p-value | OR (95% CI) | p-value | OR (95% CI) | p-value | OR (95% CI) | p-value |
| Female sex | 1.06 (0.51-2.23) | 0.872 |  |  | 3.23 (1.30-7.98) | **0.011** | 3.60 (1.22-10.65) | **0.020** |
| Age | 0.99 (0.97-1.01) | 0.297 |  |  | 1.00 (0.98-1.03) | 0.692 |  |  |
| Location prior to admission^a^ | 0.99 (0.93-1.06) | 0.840 |  |  | 1.38 (0.86-2.23) | **0.183** | 1.33 (0.69-2.55) | 0.390 |
| Ward | 0.94 (0.61-1.44) | 0.763 |  |  | 1.39 (0.81-2.37) | 0.233 |  |  |
| History of hospitalization^b^ | 1.68 (0.71-4.00) | 0.241 |  |  | 0.46 (0.15-1.42) | 0.177 |  |  |
| History of ICU stay^b^ | 1.83 (0.72-4.68) | 0.204 |  |  | 0.49 (0.17-1.37) | 0.174 |  |  |
| Hospitalization abroad^b^ | 16.24 (6.98-37.79) | **<0.001** | 16.54 (6.50-42.10) | **<0.001** | 5.45 (1.94-15.32) | **0.001** | 2.64 (0.70-10.01) | 0.152 |
| Charlson Comorbidity Index | 1.07 (0.91-1.25) | 0.429 |  |  | 1.09 (0.87-1.36) | 0.462 |  |  |
| Active open wounds | 1.28 (0.43-3.83) | 0.655 |  |  | 0.85 (0.24-2.95) | 0.793 |  |  |
| Surgery^b^ | 2.96 (1.40-6.23) | **0.004** | 1.64 (0.70-3.82) | 0.251 | 1.50 (0.63-3.58) | 0.361 |  |  |
| Urinary catheterization^c^ | 1.26 (0.57-2.75) | 0.570 |  |  | 0.70 (0.28-1.74) | 0.447 |  |  |
| Vascular hardware^d^ | 2.31 (0.50-10.67) | 0.283 |  |  | 0.70 (0.13-3.70) | 0.677 |  |  |
| Dialysis | 1.58 (0.19-12.74) | 0.670 |  |  | - |  |  |  |
| Allogeneic stem cell transplantation | 1.20 (0.15-9.53) | 0.860 |  |  | 1.09 (0.09-12.47) | 0.947 |  |  |
| History of colonization with CPB/ESBL-PE^b,e^ | 0.50 (0.21-1.19) | 0.115 |  |  | 0.22 (0.08-0.58) | **0.002** | 0.78 (0.19-3.19) | 0.735 |
| History of infection with CPB/ESBL-PE^b,e^ | 0.37 (0.09-1.60) | 0.185 |  |  | 0.19 (0.04-0.86) | **0.032** | 0.32 (0.05-2.00) | 0.222 |
| History of antibiotic therapy^f^ | 2.73 (1.23-6.10) | **0.014** | 3.46 (1.42-8.44) | **0.006** | 1.46 (0.58-3.69) | 0.425 |  |  |
| - Duration (days) | 1.01 (1.00-1.02) | 0.119 |  |  | 1.00 (1.00-1.01) | 0.372 |  |  |
| Immunosuppressive therapy^b^ | 0.97 (0.42-2.24) | 0.950 |  |  | 0.88 (0.33-2.32) | 0.797 |  |  |
| Proton pump inhibitor^f^ | 0.61 (0.29-1.29) | 0.197 |  |  | 0.28 (0.11-0.68) | **0.005** | 0.32 (0.10-0.96) | **0.041** |
| OR = odds ratio, 95%CI = 95% confidence interval, ICU = intensive care unit, CPB = carbapenemase-producing bacteria, ESBL-PE = extended-spectrum beta-lactamase-producing Enterobacterales  ^a^ home, other acute-care facility or nursing-home  ^b^ within the past 12 months  ^c^ within 30 days prior to CPB detection  ^d^ to be in place for at least 7 days prior to CPB detection  ^e^ refers to ESBL-PE and/or CPB in the CPB group and to only ESBL-PE in the ESBL-PE group  ^f^ within 3 months prior to CPB detection | | | | | | | | |

| **Supplementary table 6:** Comparison of risk-factors between patients with detection of only carbapenemases-producing Enterobacterales with detection of CPB between 01/2016-12/2018 (n=20) versus patients with detection of ESBL-PE (n=572) | | | | |
| --- | --- | --- | --- | --- |
|  | Univariable analysis | | Multivariable analysis | |
|  | OR (95% CI) | p-value | OR (95% CI) | p-value |
| Demographics and clinical data | | | | |
| Female sex | 1.60 (0.64-4.00) | 0.313 |  |  |
| Age | 1.00 (0.98-1.02) | 0.917 |  |  |
| Ward | 0.93 (0.54-1.61) | 0.791 |  |  |
| History of hospitalization^a^ | 1.33 (0.48-3.73) | 0.583 |  |  |
| History of ICU stay^a^ | 1.86 (0.66-5.24) | 0.242 |  |  |
| Hospitalisation abroad^a^ | 12.55 (4.88-32.30) | **<0.001** | 12.29 (4.55-33.21) | **<0.001** |
| Charlson Comorbidity Index | 1.08 (0.89-1.31) | 0.427 |  |  |
| Open wounds | 1.92 (0.62-5.91) | 0.257 |  |  |
| Surgery^a^ | 2.58 (1.05-6.30) | **0.038** | 1.60 (0.61-4.17) | 0.338 |
| Urinary catheterization^b^ | 0.99 (0.37-2.61) | 0.981 |  |  |
| Vascular hardware^c^ | 2.78 (0.61-12.72) | 0.188 |  |  |
| Dialysis | 2.96 (0.36-24.30) | 0.313 |  |  |
| Allogeneic stem cell transplantation | 1.83 (0.23-14.51) | 0.568 |  |  |
| History of colonization with ESBL-PE/CPB^a,d^ | 0.47 (0.17-1.31) | 0.150 |  |  |
| History of infection with ESBL-PE/CPB^a,d^ | 0.25 (0.03-1.90) | 0.181 |  |  |
| History of antibiotic therapy^e^ | 2.45 (0.93-6.47) | **0.070** | 2.84 (1.02-7.86) | **0.045** |
| Duration of antibiotic therapy (days) | 1.01 (1.00-1.02) | 0.127 |  |  |
| Immunosuppressive therapy^a^ | 1.16 (0.44-3.08) | 0.761 |  |  |
| Proton pump inhibitor^e^ | 0.54 (0.22-1.33) | 0.179 |  |  |
| OR = odds ratio, 95%CI = 95% confidence interval, ICU = intensive care unit, CPB = carbapenemases-producing bacteria, ESBL-PE = extended-spectrum beta-lactamase-producing Enterobacterales | | | | |
| ^a^ within the past 12 months  ^b^ within 30 days prior to CPB detection  ^c^ to be in place for at least 7 days prior to CPB detection  ^d^ refers to ESBL-PE and/or CPB in the CPB group and to only ESBL-PE in the ESBL-PE group  ^e^ within 3 months prior to CPB detection | | | | |

| **Supplementary table 7:** Subgroup analysis of patients with detection of only CP-Enterobacterales with CPB-detection between 01/2016-12/2018 (n=20) versus patients with detection of only ESBL-*E.coli* (n=467) **(A)** and versus patients with detection of only ESBL-*K. pneumoniae* (n=65) **(B)** | | | | | | | | |
| --- | --- | --- | --- | --- | --- | --- | --- | --- |
|  | **(A)** | | | | **(B)** | | | |
|  | Univariable analyses | | Multivariable analyses | | Univariable analyses | | Multivariable analyses | |
|  | OR (95% CI) | p-value | OR (95% CI) | p-value | OR (95% CI) | p-value | OR (95% CI) | p-value |
| Female sex | 1.39 (0.56-3.47) | 0.475 |  |  | 4.24 (1.48-12.12) | **0.007** | 5.47 (1.54-19.42) | **0.009** |
| Age | 1.00 (0.97-1.02) | 0.727 |  |  | 1.01 (0.98-1.04) | 0.435 |  |  |
| Location prior to admission^a^ | 1.00 (0.91-1.08) | 0.848 |  |  | 1.30 (0.75-2.27) | 0.345 |  |  |
| Ward | 0.89 (0.51-1.54) | 0.676 |  |  | 1.49 (0.72-3.08) | 0.286 |  |  |
| History of hospitalization^b^ | 1.53 (0.55-4.30) | 0.416 |  |  | 0.42 (0.12-1.48) | 0.176 |  |  |
| History of ICU stay^b^ | 2.45 (0.86-7.00) | 0.095 |  |  | 0.65 (0.21-2.03) | 0.459 |  |  |
| Hospitalization abroad^b^ | 17.38 (6.50-46.47) | **<0.001** | 18.54 (6.29-54.63) | **<0.001** | 5.83 (1.84-18.42) | **0.003** | 4.40 (1.04-18.62) | **0.044** |
| Charlson Comorbidity Index | 1.07 (0.89-1.30) | 0.453 |  |  | 1.10 (0.85-1.42) | 0.464 |  |  |
| Active open wounds | 2.09 (0.67-6.48) | 0.204 |  |  | 1.38 (0.38-4.98) | 0.627 |  |  |
| Surgery^b^ | 2.96 (1.20-7.28) | **0.018** | 1.73 (0.63-4.70) | 0.285 | 1.50 (0.55-4.11) | 0.430 |  |  |
| Urinary catheterization^c^ | 1.08 (0.41-2.86) | 0.883 |  |  | 0.60 (0.21-1.77) | 0.357 |  |  |
| Vascular hardware^d^ | 3.60 (0.76-17.02) | 0.107 |  |  | 1.09 (0.20-5.89) | 0.918 |  |  |
| Dialysis | 2.41 (0.29-19.76) | 0.414 |  |  | - |  |  |  |
| Allogeneic stem cell transplantation | 1.84 (0.23-14.79) | 0.567 |  |  | 1.66 (0.14-19.30) | 0.686 |  |  |
| History of colonization with CPB/ESBL-PE^b,e^ | 0.55 (0.20-1.53) | 0.249 |  |  | 0.24 (0.08-0.73) | **0.012** | 0.70 (0.17-2.88) | 0.626 |
| History of infection with CPB/ESBL-PE^b,e^ | 0.28 (0.04-2.09) | 0.212 |  |  | 0.14 (0.02-1.10) | 0.062 |  |  |
| History of antibiotic therapy^f^ | 2.73 (1.03-7.24) | **0.043** | 3.78 (1.28-11.11) | **<0.001** | 1.46 (0.50-4.29) | 0.493 |  |  |
| - Duration (days) | 1.01 (1.00-1.03) | 0.099 |  |  | 1.00 (1.00-1.01) | 0.278 |  |  |
| Immunosuppressive therapy^b^ | 1.15 (0.43-3.05) | 0.783 |  |  | 1.04 (0.35-3.10) | 0.947 |  |  |
| Proton pump inhibitor^f^ | 0.61 (0.24-1.52) | 0.287 |  |  | 0.28 (0.10-0.78) | **0.015** | 0.21 (0.06-0.76) | **0.018** |
| OR = odds ratio, 95%CI = 95% confidence interval, ICU = intensive care unit, CPB = carbapenemase-producing bacteria, ESBL-PE = extended-spectrum beta-lactamase-producing Enterobacterales  ^a^ home, other acute-care facility or nursing-home  ^b^ within the past 12 months  ^c^ within 30 days prior to CPB detection  ^d^ to be in place for at least 7 days prior to CPB detection  ^e^ refers to ESBL-PE and/or CPB in the CPB group and to only ESBL-PE in the ESBL-PE group  ^f^ within 3 months prior to CPB detection | | | | | | | | |

| **Supplementary table 8:** International travel destinations of the CPB travel subgroup | |
| --- | --- |
| **N** | **Country** |
| 7 | Serbia |
| 3 | Italy |
| 3 | Turkey |
| 2 | Dominican Republic |
| 2 | Spain (incl. Canary islands) |
| 2 | Thailand |
| 1 | Brazil |
| 1 | Colombia |
| 1 | Egypt |
| 1 | Estonia |
| 1 | Greece |
| 1 | Hungary |
| 1 | India |
| 1 | Lebanon |
| 1 | Marocco |
| 1 | North Macedonia |
| 1 | Russia |
| 1 | Sri Lanka |
